# Supplementary material for: The planarian wound epidermis gene equinox is required for blastema formation in regeneration
Source: Nat Commun. 2022 May 18;13:2726. doi: 10.1038/s41467-022-30412-6 (PMC9117669; doi:10.1038/s41467-022-30412-6)
Supplement: Supplementary file 1 — Supplementary Information [file 41467_2022_30412_MOESM1_ESM.pdf]

The planarian wound epidermis gene *equinox* is required for blastema formation in regeneration

M. Lucila Scimone[1,2,†], Jennifer K. Cloutier[2,3,4,†] Chloe L. Maybrun[2,3], and Peter W. Reddien[1,2,3,*]

Nature Communications

Supplementary Information

Supplementary Figures 1-14
Supplementary Data 1-3

Supplementary Data

Supplementary Data 1. Differential expression analysis of regenerating control and *bmp4* RNAi animals.

Supplementary Data 2. Accession numbers of proteins with similar structure analyzed in the phylogenetic tree.

Supplementary Data 3. Differential expression analysis of regenerating control and *equinox* RNAi animals.

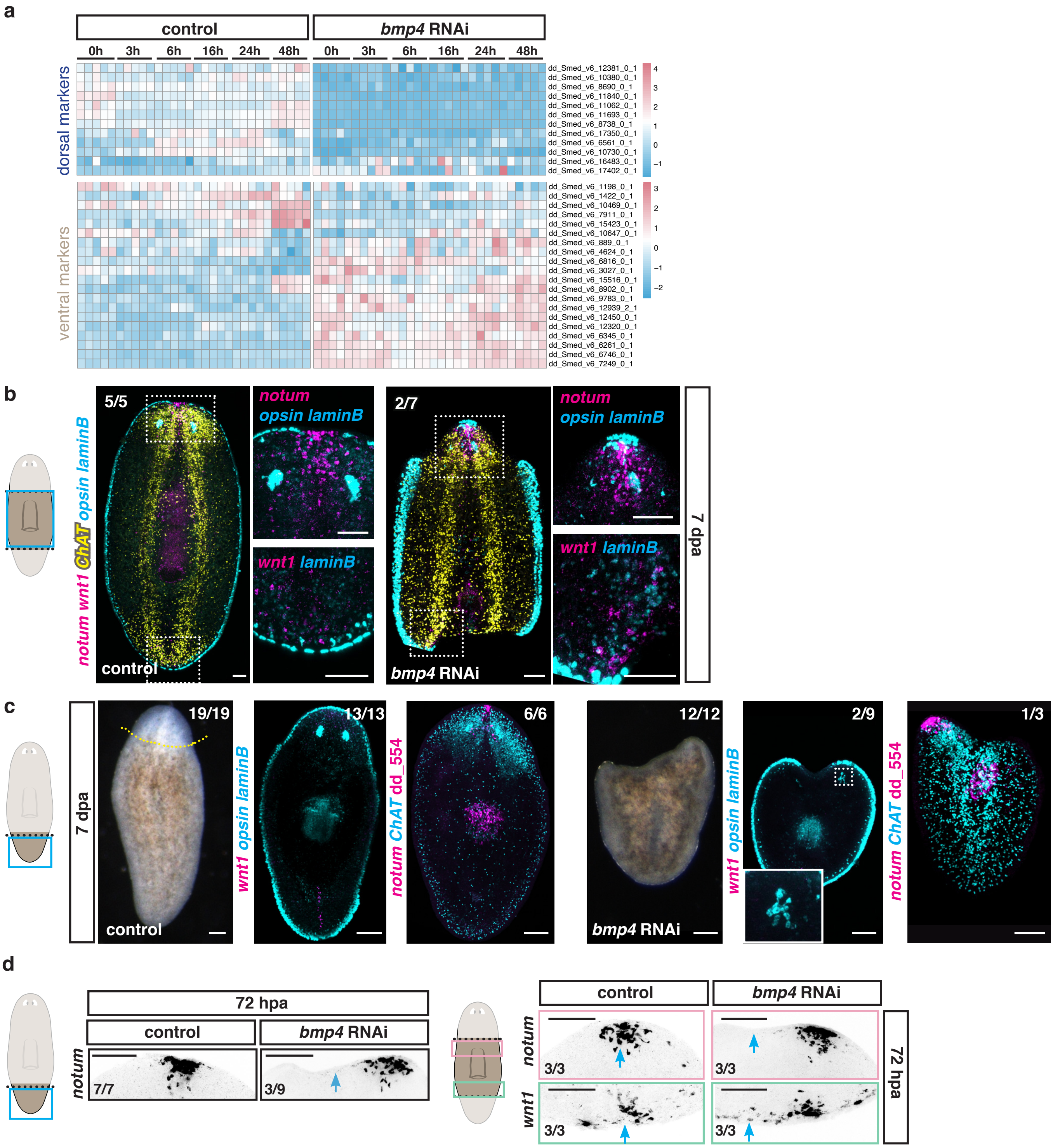
a
control
bmp4 RNAi
0h 3h 6h 16h 24h 48h
dorsal markers
ventral markers
b
notum wnt1 ChAT opsin laminB
5/5
control
notum opsin laminB
wnt1 laminB
2/7
bmp4 RNAi
7 dpa
c
7 dpa
19/19
control
wnt1 opsin laminB
13/13
notum ChAT dd_554
6/6
12/12
bmp4 RNAi
2/9
1/3
d
72 hpa
control
bmp4 RNAi
notum
7/7
3/9
wnt1
3/3

Figure S1

Supplementary Figure 1. *bmp4* is required for planarian regeneration. (a) Heatmap shows ventralization in *bmp4* RNAi animals. (b) *bmp4* RNAi animals do not regenerate a blastema. No *laminB*$^+$ DVB epidermal cells are regenerated after *bmp4* RNAi (5/7). Occasionally, a new anterior pole forms at a new DVB patch of cells facilitating formation of eye cells in *bmp4* RNAi animals (2/7). Posterior poles form asymmetrically at the pre-existing DVB in *bmp4* RNAi animals. Data are representative of two independent experiments. (c) Occasionally a new anterior pole forms asymmetrically at the pre-existing DVB of a tail fragment after *bmp4* RNAi, facilitating the nucleation of eye cells and pharynx. Data are representative of at least two independent experiments. (d) Anterior pole forms in trunk fragments (right) and occasionally in tail fragments (left) of *bmp4* RNAi animals but only asymmetrically. Data are representative of two independent experiments. Blue arrows, pre-existing midline. Dotted yellow line marks amputation plane. Box in the cartoon shows the location of the image taken. Dotted box, zoom in area. Scale bars, 100 μm.

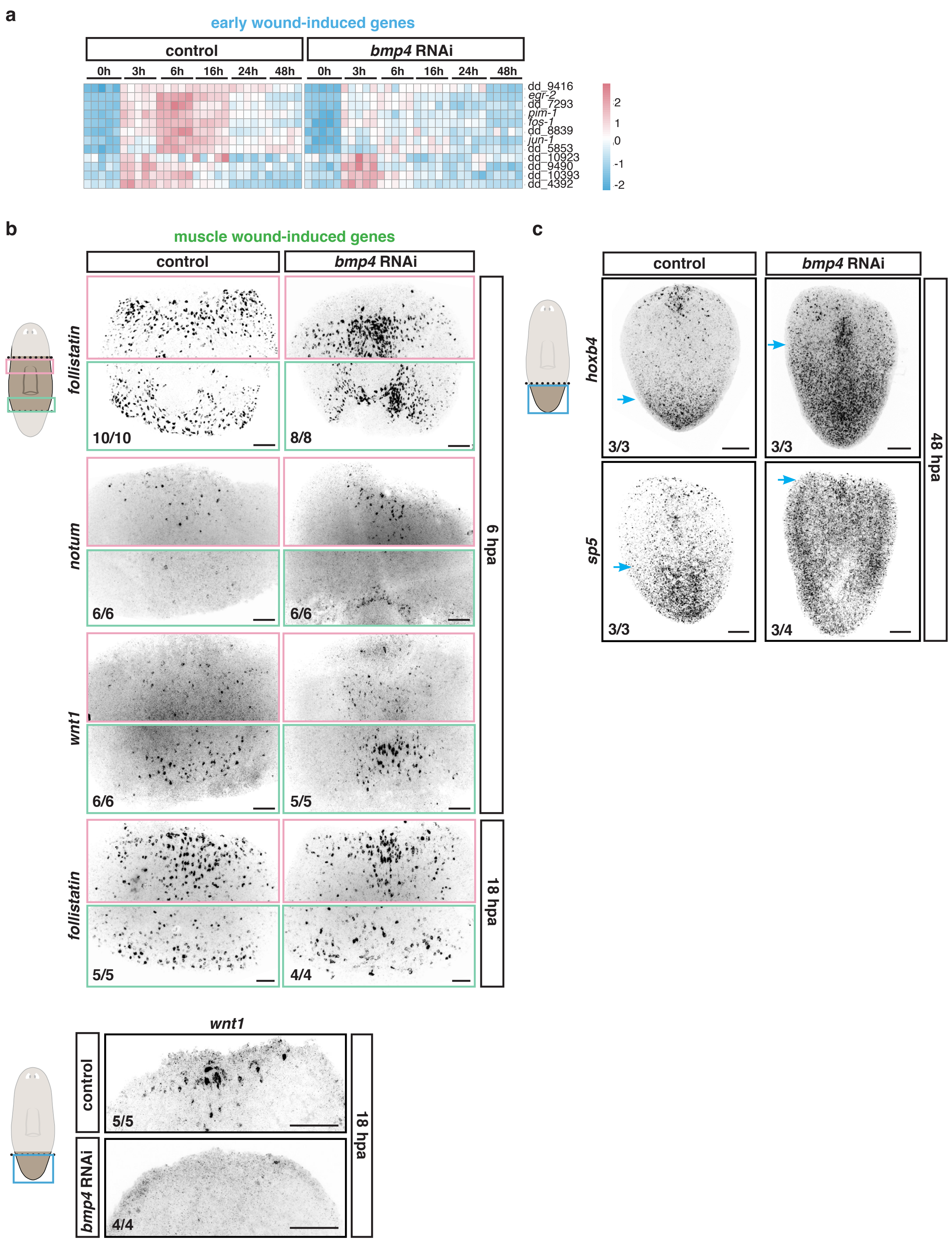

a
early wound-induced genes
control
bmp4 RNAi
0h
3h
6h
16h
24h
48h
dd_9416
egr-2
dd_7293
pim-1
fos-1
dd_8839
jun-1
dd_5853
dd_10923
dd_9490
dd_10393
dd_4392
2
1
0
-1
-2
b
muscle wound-induced genes
control
bmp4 RNAi
follistatin
10/10
8/8
notum
6/6
6/6
wnt1
6/6
5/5
6 hpa
follistatin
5/5
4/4
18 hpa
wnt1
control
5/5
bmp4 RNAi
4/4
18 hpa
c
control
bmp4 RNAi
hoxb4
3/3
3/3
sp5
3/3
3/4
48 hpa

Figure S2

Supplementary Figure 2. *bmp4* is required for maintenance of wound-induced gene expression and PCG rescaling. (a) Heatmap shows activation of early wound-induced genes in *bmp4* RNAi animals. (b) Activation but not maintenance of wound-induced gene expression in *bmp4* RNAi animals. (c) No rescaling of posterior PCGs in regenerating tail fragments after *bmp4* RNAi. Data shown in (b, c) are representative of two independent experiments. Blue arrows, anterior expression edge of the posterior PCG. Box in the cartoon shows the location of the image taken. Scale bars, 100 μm.

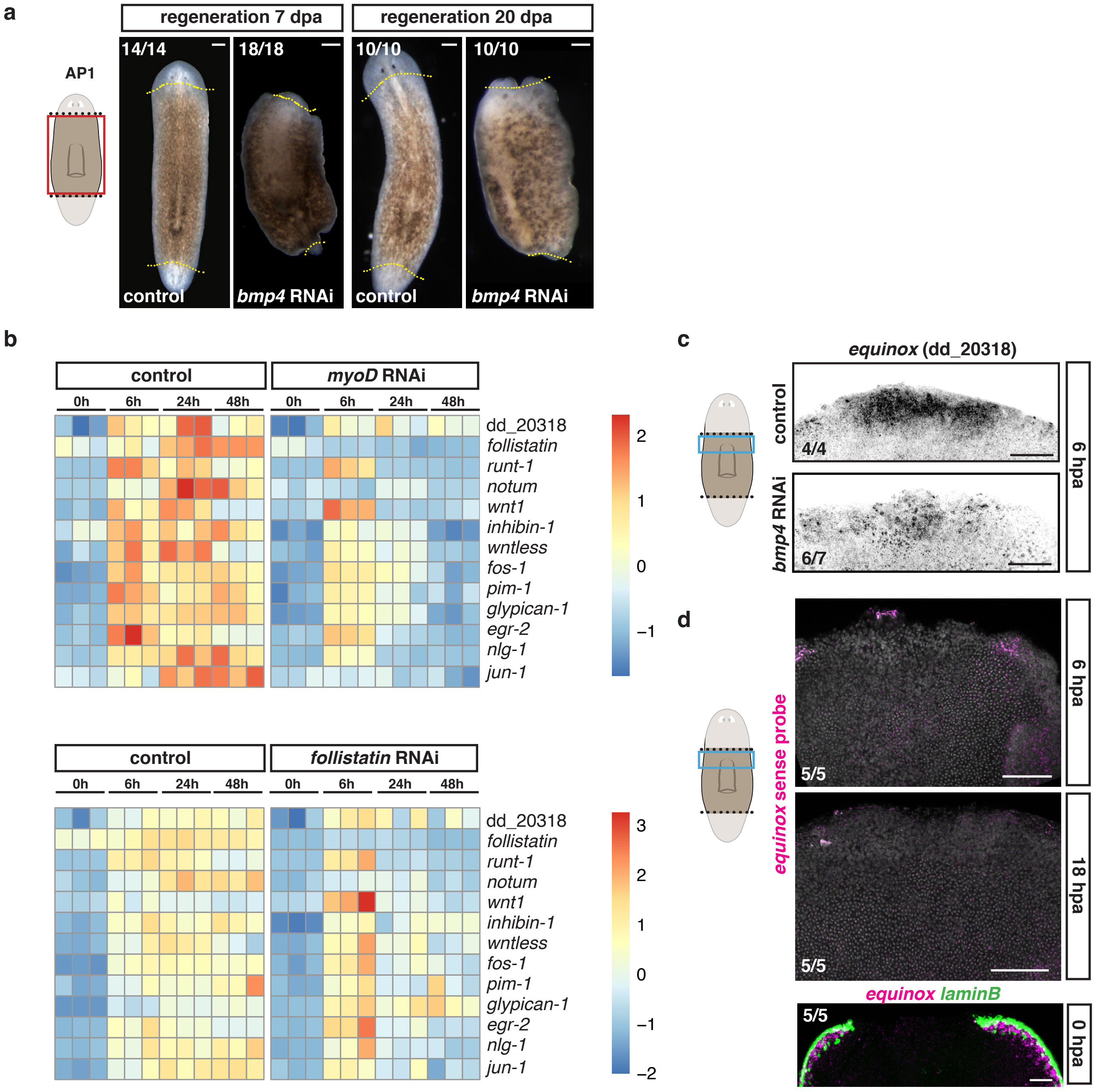

a
regeneration 7 dpa
regeneration 20 dpa
AP1
14/14
18/18
10/10
10/10
control
bmp4 RNAi
control
bmp4 RNAi
b
control
myoD RNAi
0h
6h
24h
48h
dd_20318
follistatin
runt-1
notum
wnt1
inhibin-1
wntless
fos-1
pim-1
glypican-1
egr-2
nlg-1
jun-1
2
1
0
−1
control
follistatin RNAi
3
2
1
0
−1
−2
c
equinox (dd_20318)
control
bmp4 RNAi
4/4
6/7
6 hpa
d
equinox sense probe
5/5
6 hpa
5/5
18 hpa
equinox laminB
5/5
0 hpa

Figure S3

Supplementary Figure 3. Expression of wound-induced *equinox* is inhibited in *bmp4* but not in *myoD* or *follistatin* RNAi. (a) No regeneration of *bmp4* RNAi animals after AP1 cuts. Dotted yellow line marks amputation plane. (b) Heatmaps show wound-induced gene expression in *myoD* and *follistatin* RNAi animals. (c) Wound-induced *equinox* expression is not activated after *bmp4* RNAi (top). (d) Control expression of *equinox* after wounding. Sense probe for *equinox* does not show wound-induced expression following amputation (top). No *equinox* expression is detected at 0 hpa. Results shown in (a, c, and d) are representative of two independent experiments. Scale bars, 100 μm.

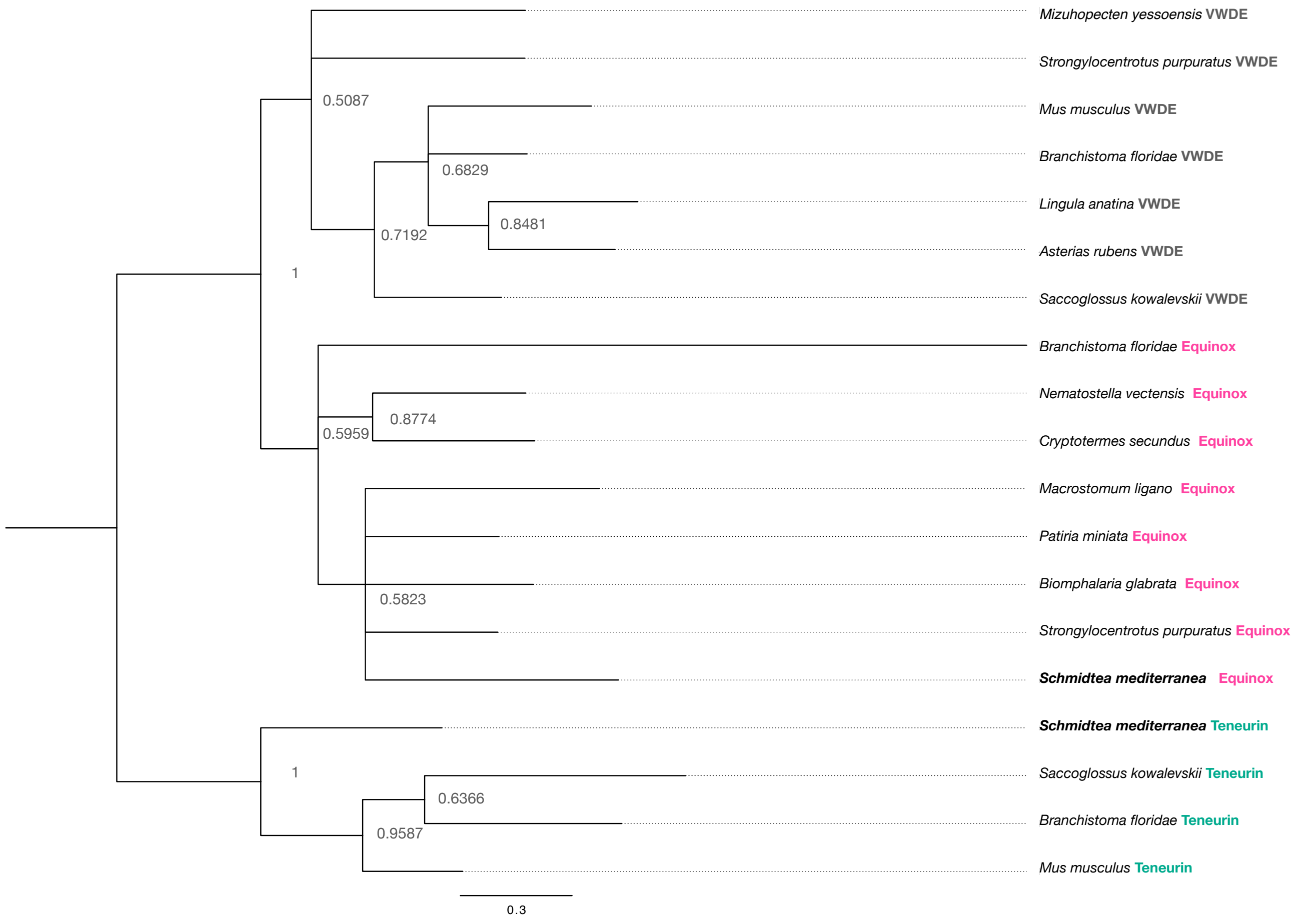
Mizuhopecten yessoensis VWDE
Strongylocentrotus purpuratus VWDE
0.5087
Mus musculus VWDE
Branchistoma floridae VWDE
0.6829
Lingula anatina VWDE
0.8481
0.7192
Asterias rubens VWDE
1
Saccoglossus kowalevskii VWDE
Branchistoma floridae Equinox
Nematostella vectensis Equinox
0.8774
0.5959
Cryptotermes secundus Equinox
Macrostomum ligano Equinox
Patiria miniata Equinox
Biomphalaria glabrata Equinox
0.5823
Strongylocentrotus purpuratus Equinox
Schmidtea mediterranea Equinox
Schmidtea mediterranea Teneurin
1
Saccoglossus kowalevskii Teneurin
0.6366
Branchistoma floridae Teneurin
0.9587
Mus musculus Teneurin
0.3

Figure S4

Supplementary Figure 4. Phylogenetic tree for Equinox and VWDE. Phylogenetic tree for the placement of *Schmidtea mediterranea* Equinox. Bayesian analysis of the EGF domain containing portion of proteins. This tree compares Equinox to best blast hit VWDE, where Teneurin is used as an outgroup across species. Percent posterior probability is indicated at nodes. Protein sequences are provided in Supplementary Table 2.

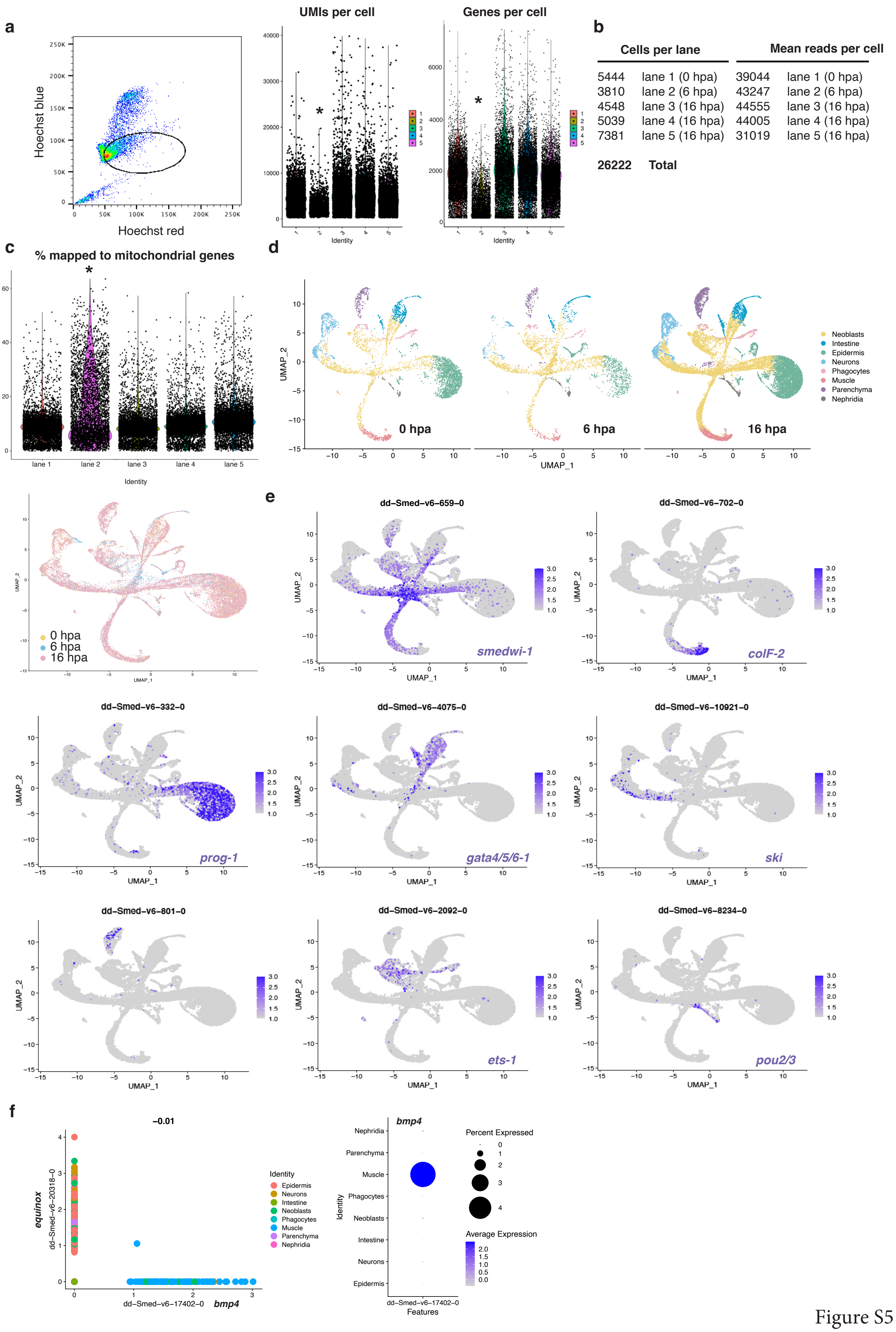
a
Hoechst blue
Hoechst red
UMIs per cell
Genes per cell
Identity
b
Cells per lane
Mean reads per cell
5444 lane 1 (0 hpa) 39044 lane 1 (0 hpa)
3810 lane 2 (6 hpa) 43247 lane 2 (6 hpa)
4548 lane 3 (16 hpa) 44555 lane 3 (16 hpa)
5039 lane 4 (16 hpa) 44005 lane 4 (16 hpa)
7381 lane 5 (16 hpa) 31019 lane 5 (16 hpa)
26222 Total
c
% mapped to mitochondrial genes
lane 1
lane 2
lane 3
lane 4
lane 5
Identity
d
0 hpa
6 hpa
16 hpa
UMAP_1
UMAP_2
Neoblasts
Intestine
Epidermis
Neurons
Phagocytes
Muscle
Parenchyma
Nephridia
e
dd–Smed–v6–659–0
smedwi-1
dd–Smed–v6–702–0
colF-2
dd–Smed–v6–332–0
prog-1
dd–Smed–v6–4075–0
gata4/5/6-1
dd–Smed–v6–10921–0
ski
dd–Smed–v6–801–0
dd–Smed–v6–2092–0
ets-1
dd–Smed–v6–8234–0
pou2/3
f
−0.01
equinox
dd–Smed–v6–20318–0
dd–Smed–v6–17402–0
bmp4
Identity
Epidermis
Neurons
Intestine
Neoblasts
Phagocytes
Muscle
Parenchyma
Nephridia
Percent Expressed
Average Expression
Features

Figure S5

Supplementary Figure 5. *equinox* is expressed in epidermal cells. (a) Graphs show number of UMIs (left) and genes (right) per cell in each of the sequencing lanes. *, lane 2 cells from an early wounded time point were sorted including calcein intermediate assuming some wounded cells might have a compromised membrane. Likely for this reason the overall quality of lane 2 cells was lower (see part c). (b) Tables show total number of cells per lane, and mean reads per cell. (c) Violin plots show percentage of mapping to mitochondrial genes. *, lane 2 cells from an early wounded time point were sorted including calcein intermediate assuming wounded cells might have compromised membrane. Likely for this reason, cells displayed higher percentage of reads that mapped to mitochondrial genes, reflecting more permeable cells. Despite this caveat, these cells still displayed non overlapping cell type-specific gene expression and were utilized for data analysis that was validated by FISH. (d) UMAP plot shows the distribution of cells from each time point (left). UMAP plot shows contribution of cells to different clusters per time point (right). (e) UMAP plots show expression of specific tissue markers: *smedwi-1*, neoblasts; *prog-1,* epidermal progenitors; *colF*-2, muscle; *gata4/5/6-1,* intestine; *ets-1,* phagocytes; dd_801 (no match), parenchymal cells; *pou2/3,* protonephridia; *ski,* neurons. (f) No correlation between *bmp4* and *equinox* expression.

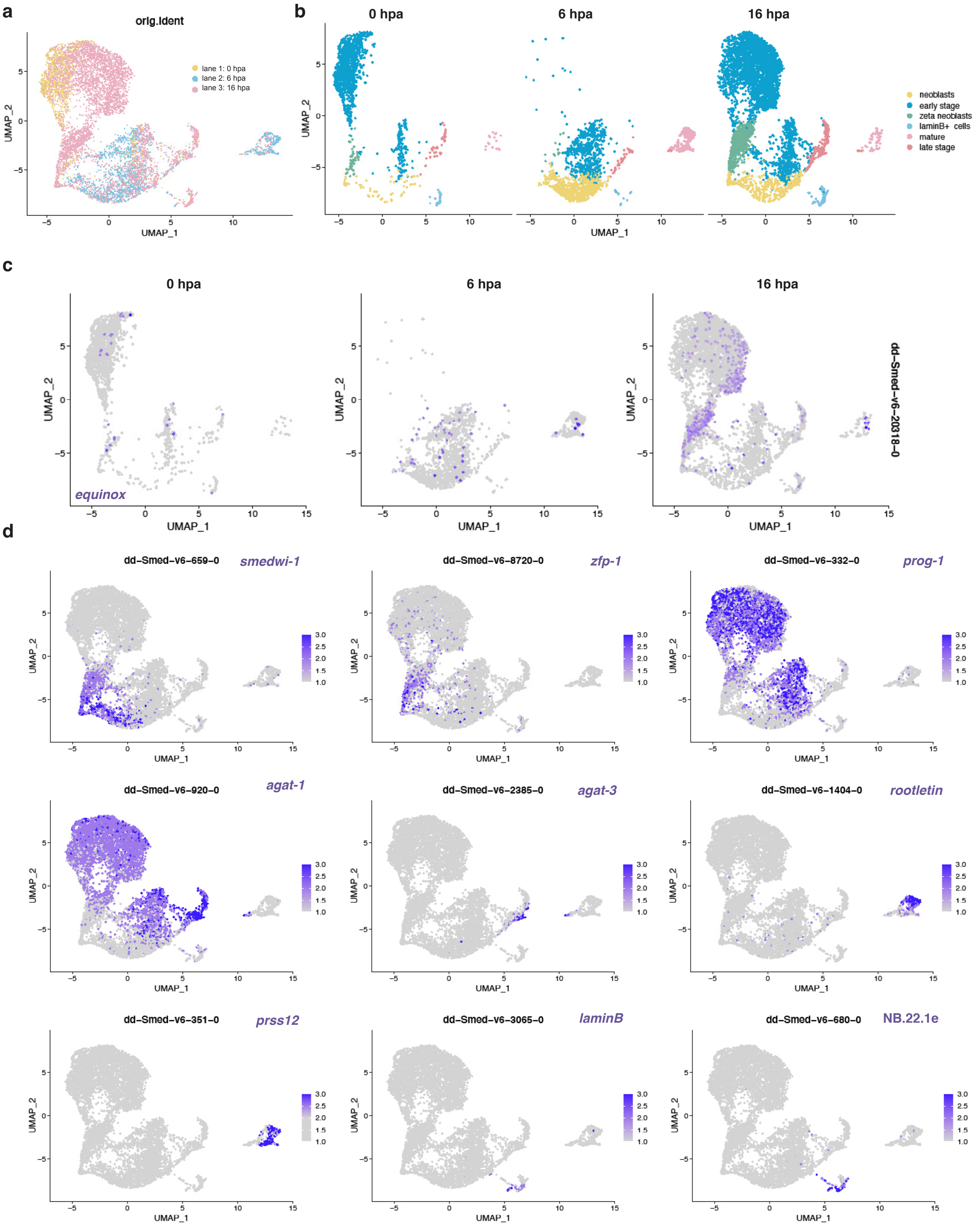

a
orig.ident
lane 1: 0 hpa
lane 2: 6 hpa
lane 3: 16 hpa
UMAP_1
UMAP_2
b
0 hpa
6 hpa
16 hpa
neoblasts
early stage
zeta neoblasts
laminB+ cells
mature
late stage
c
equinox
dd–Smed–v6–20318–0
d
dd–Smed–v6–659–0
smedwi-1
dd–Smed–v6–8720–0
zfp-1
dd–Smed–v6–332–0
prog-1
dd–Smed–v6–920–0
agat-1
dd–Smed–v6–2385–0
agat-3
dd–Smed–v6–1404–0
rootletin
dd–Smed–v6–351–0
prss12
dd–Smed–v6–3065–0
laminB
dd–Smed–v6–680–0
NB.22.1e

Figure S6

Supplementary Figure 6. All different stages through the epidermal differentiation lineage are represented in the single-cell RNA sequencing data. (a) UMAP plot shows distribution of cells along the epidermal differentiation pathway collected at different time points following amputation. (b) UMAP plot shows distribution of cells within the epidermal lineage from each time point. (c) UMAP plot shows *equinox* expression in each time point. (d) UMAP plots show expression of different epidermal differentiation markers: *smedwi-1,* neoblasts; *zfp-1,* zeta-neoblasts; *prog-1,* early progenitors; *agat-1,* early and late progenitors; *agat-3,* late progenitors; *rootletin,* mature epidermis; *prss12,* mature epidermis; *laminB,* mature DVB epidermis; NB.22.1e, mature DVB epidermis.

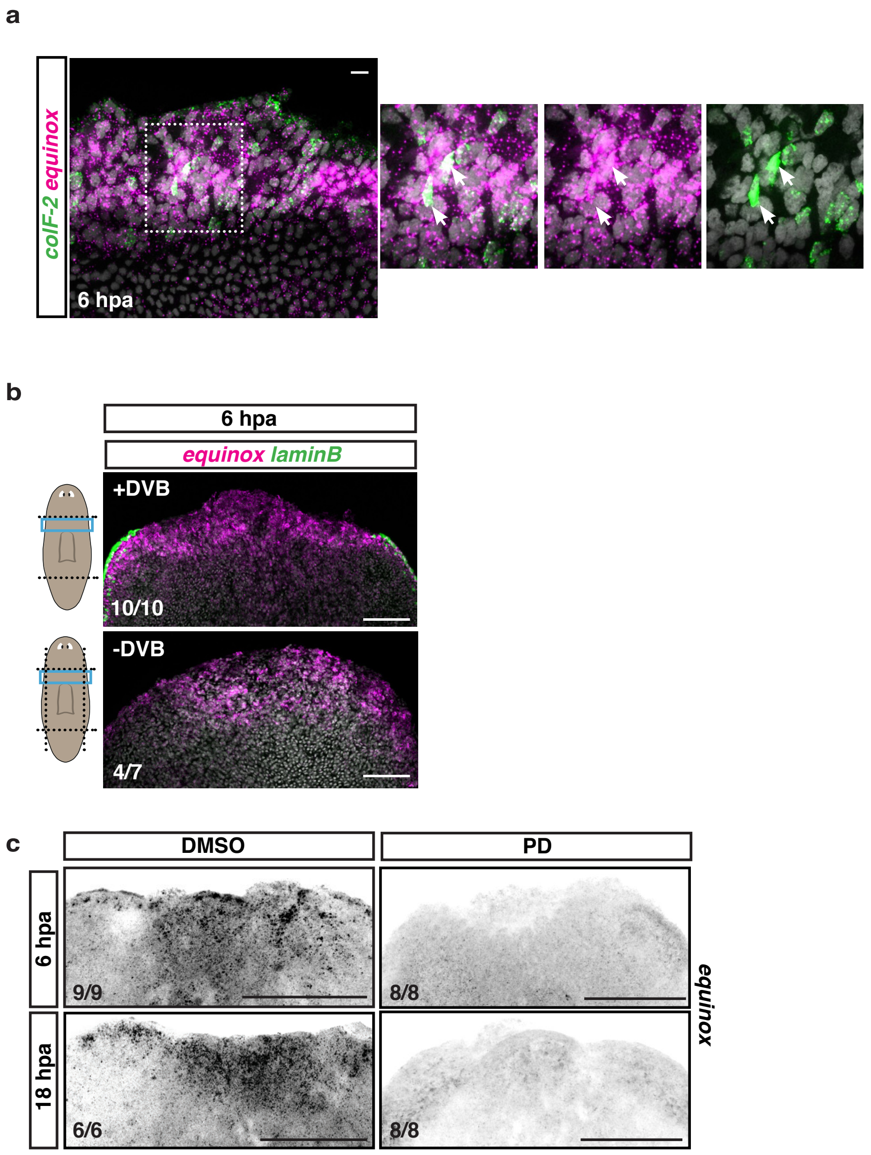
a
colF-2 equinox
6 hpa
b
6 hpa
equinox laminB
+DVB
10/10
-DVB
4/7
c
DMSO
PD
6 hpa
18 hpa
9/9
8/8
6/6
8/8
equinox

Figure S7

Supplementary Figure 7. Wound-induced *equinox* cells are not only pre-existing DVB cells. (a) Rare muscle cells expressed *equinox* following wounding at 6 hpa, n=2 independent experiments. (b) Wound-induced *equinox* expression is not the result of migration of DVB *equinox*$^+$ cells, n=3 independent experiments. (c) *equinox* expression at both 6 and 18 hpa is reduced following inhibition of Erk signaling, n=2 independent experiments. Scale bar, 100 µm.

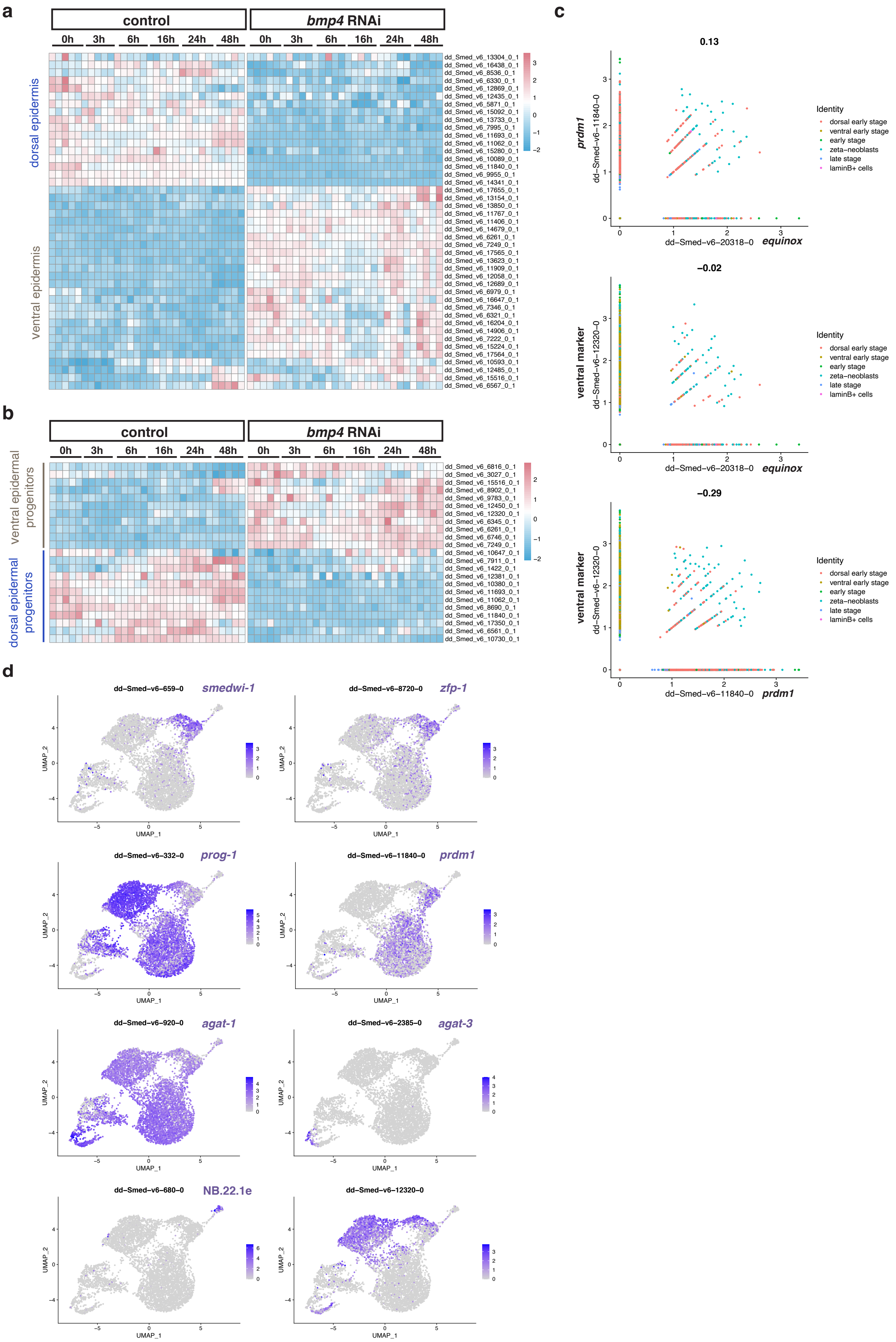

a
control
bmp4 RNAi
0h
3h
6h
16h
24h
48h
dorsal epidermis
ventral epidermis
dd_Smed_v6_13304_0_1
dd_Smed_v6_16438_0_1
dd_Smed_v6_8536_0_1
dd_Smed_v6_6330_0_1
dd_Smed_v6_12869_0_1
dd_Smed_v6_12435_0_1
dd_Smed_v6_5871_0_1
dd_Smed_v6_15092_0_1
dd_Smed_v6_13733_0_1
dd_Smed_v6_7995_0_1
dd_Smed_v6_11693_0_1
dd_Smed_v6_11062_0_1
dd_Smed_v6_15280_0_1
dd_Smed_v6_10089_0_1
dd_Smed_v6_11840_0_1
dd_Smed_v6_9955_0_1
dd_Smed_v6_14341_0_1
dd_Smed_v6_17655_0_1
dd_Smed_v6_13154_0_1
dd_Smed_v6_13850_0_1
dd_Smed_v6_11767_0_1
dd_Smed_v6_11406_0_1
dd_Smed_v6_14679_0_1
dd_Smed_v6_6261_0_1
dd_Smed_v6_7249_0_1
dd_Smed_v6_17565_0_1
dd_Smed_v6_13623_0_1
dd_Smed_v6_11909_0_1
dd_Smed_v6_12058_0_1
dd_Smed_v6_12689_0_1
dd_Smed_v6_6979_0_1
dd_Smed_v6_16647_0_1
dd_Smed_v6_7346_0_1
dd_Smed_v6_6321_0_1
dd_Smed_v6_16204_0_1
dd_Smed_v6_14906_0_1
dd_Smed_v6_7222_0_1
dd_Smed_v6_15224_0_1
dd_Smed_v6_17564_0_1
dd_Smed_v6_10593_0_1
dd_Smed_v6_12485_0_1
dd_Smed_v6_15516_0_1
dd_Smed_v6_6567_0_1
b
control
bmp4 RNAi
0h
3h
6h
16h
24h
48h
ventral epidermal progenitors
dorsal epidermal progenitors
dd_Smed_v6_6816_0_1
dd_Smed_v6_3027_0_1
dd_Smed_v6_15516_0_1
dd_Smed_v6_8902_0_1
dd_Smed_v6_9783_0_1
dd_Smed_v6_12450_0_1
dd_Smed_v6_12320_0_1
dd_Smed_v6_6345_0_1
dd_Smed_v6_6261_0_1
dd_Smed_v6_6746_0_1
dd_Smed_v6_7249_0_1
dd_Smed_v6_10647_0_1
dd_Smed_v6_7911_0_1
dd_Smed_v6_1422_0_1
dd_Smed_v6_12381_0_1
dd_Smed_v6_10380_0_1
dd_Smed_v6_11693_0_1
dd_Smed_v6_11062_0_1
dd_Smed_v6_8690_0_1
dd_Smed_v6_11840_0_1
dd_Smed_v6_17350_0_1
dd_Smed_v6_6561_0_1
dd_Smed_v6_10730_0_1
c
0.13
prdm1
dd–Smed–v6–11840–0
dd–Smed–v6–20318–0
equinox
-0.02
ventral marker
dd–Smed–v6–12320–0
dd–Smed–v6–20318–0
equinox
-0.29
ventral marker
dd–Smed–v6–12320–0
dd–Smed–v6–11840–0
prdm1
Identity
dorsal early stage
ventral early stage
early stage
zeta–neoblasts
late stage
laminB+ cells
d
dd–Smed–v6–659–0
smedwi-1
dd–Smed–v6–8720–0
zfp-1
dd–Smed–v6–332–0
prog-1
dd–Smed–v6–11840–0
prdm1
dd–Smed–v6–920–0
agat-1
dd–Smed–v6–2385–0
agat-3
dd–Smed–v6–680–0
NB.22.1e
dd–Smed–v6–12320–0
UMAP_1
UMAP_2

Figure S8

Supplementary Figure 8. *equinox* is expressed in dorsal epidermal progenitors and these are decreased in *bmp4* RNAi animals. (a) Heatmap shows decreased expression of dorsal epidermal markers and increased expression of ventral epidermal markers after *bmp4* RNAi. (b) Heatmap shows decreased expression of dorsal early epidermal progenitor markers and increased expression of ventral early epidermal progenitor markers after *bmp4* RNAi. (c) Correlation plots showing positive correlation between *equinox* and *prdm1* (dorsal epidermal progenitor marker) and negative correlation between *equinox* and the ventral early epidermal progenitor marker dd_12320 (no match). (d) UMAP plots show expression of *smedwi-1,* neoblasts; *zfp-1,* zeta-neoblasts; *prog-1,* early progenitors; *prdm1,* dorsal early progenitors; dd_12320, ventral early progenitors; *agat-1,* early and late progenitors; *agat-3,* late progenitors, and NB.22.1e, mature DVB epidermis. Scale bar, 100 μm.

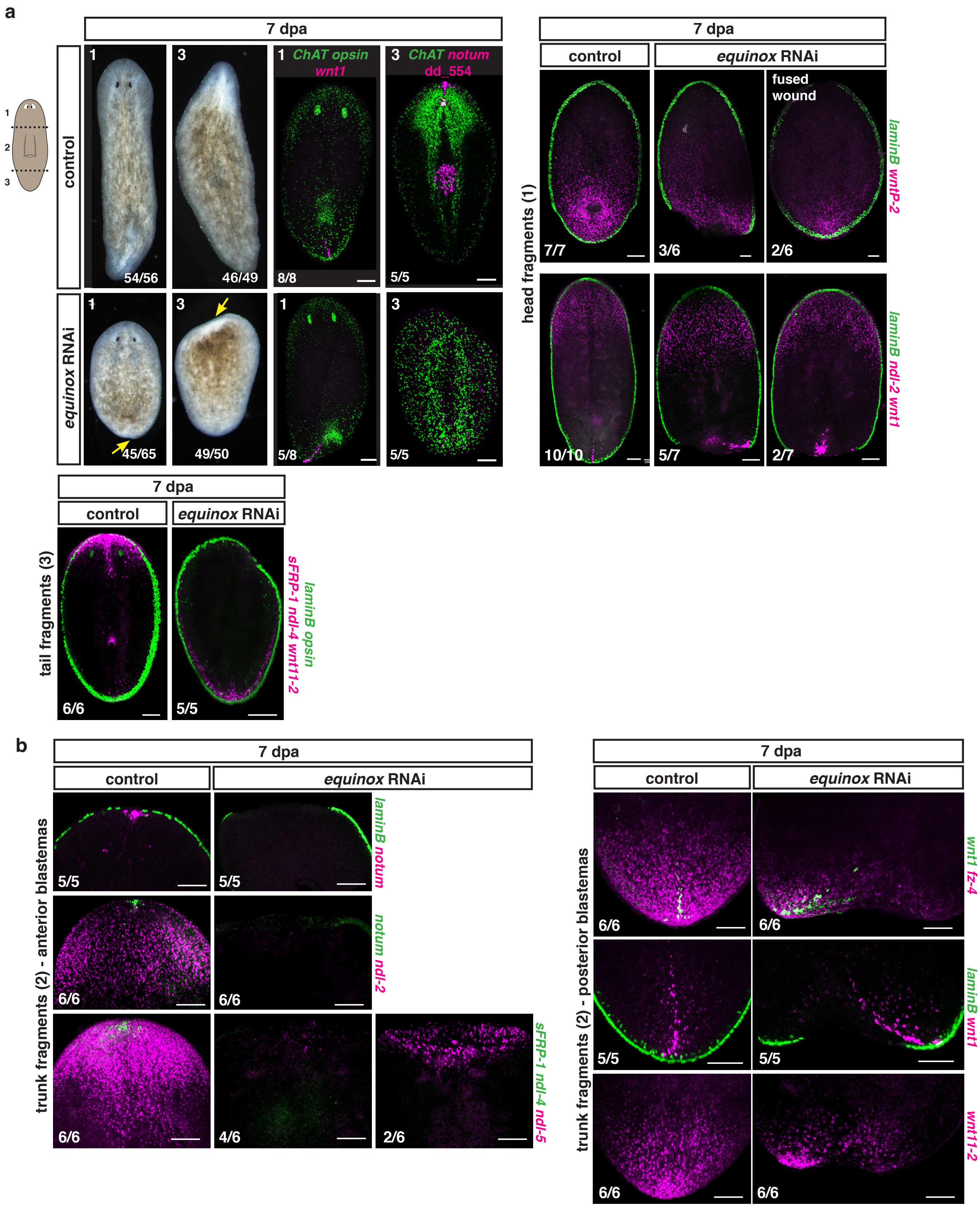
a
7 dpa
control
equinox RNAi
1
2
3
1 ChAT opsin wnt1
3 ChAT notum dd_554
54/56
46/49
8/8
5/5
45/65
49/50
5/8
5/5
head fragments (1)
fused wound
laminB wntP-2
laminB ndl-2 wnt1
7/7
3/6
2/6
10/10
5/7
2/7
tail fragments (3)
laminB opsin
sFRP-1 ndl-4 wnt11-2
6/6
5/5
b
trunk fragments (2) - anterior blastemas
laminB notum
notum ndl-2
sFRP-1 ndl-4 ndl-5
5/5
5/5
6/6
6/6
6/6
4/6
2/6
trunk fragments (2) - posterior blastemas
wnt1 fz-4
laminB wnt1
wnt11-2
6/6
6/6
5/5
5/5
6/6
6/6

Figure S9

Supplementary Figure 9. *equinox* is required for planarian regeneration. (a) *equinox* RNAi head fragments do not form a blastema, but can form an asymmetric posterior pole. *equinox* RNAi tail fragments do not form a blastema, do not form an anterior pole, do not express anterior PCGs, and do not form anterior tissues. (b) *equinox* RNAi trunk fragments do not form anterior blastemas, do not express anterior PCGs, do not form a new anterior pole, or DVB. *equinox* RNAi trunk fragments do not form posterior blastemas but are able to regenerate an asymmetric posterior pole at the pre-existing DVB and express some posterior PCGs around the posterior pole. Live images are representative of seven independent experiments. FISH images are representative of two independent experiments. Scale bars, 100 μm.

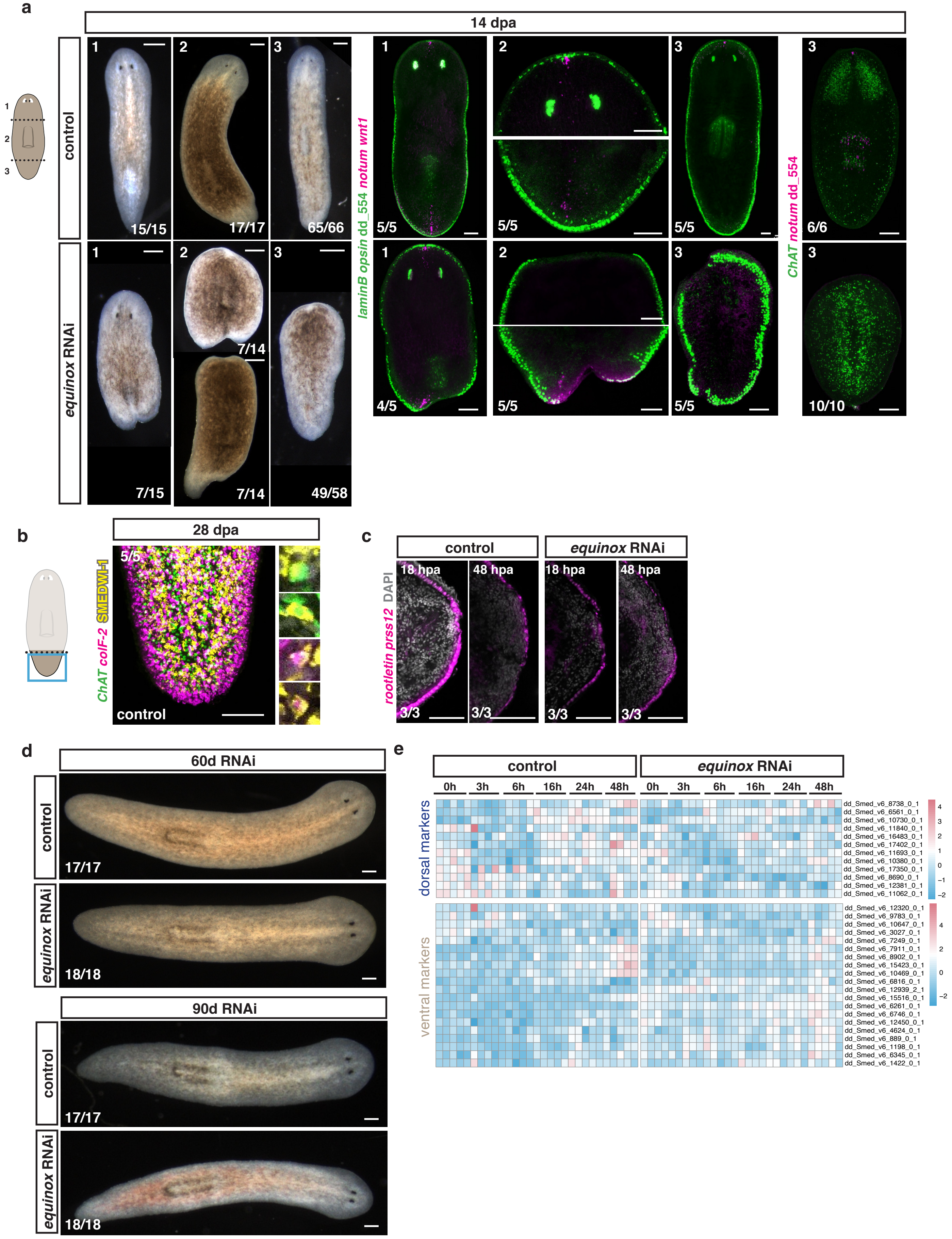
a
14 dpa
control
equinox RNAi
15/15
17/17
65/66
7/15
7/14
7/14
49/58
laminB opsin dd_554 notum wnt1
5/5
5/5
5/5
4/5
5/5
5/5
ChAT notum dd_554
6/6
10/10
b
28 dpa
ChAT colF-2 SMEDWI-1
5/5
control
c
control
equinox RNAi
18 hpa
48 hpa
18 hpa
48 hpa
rootletin prss12 DAPI
3/3
3/3
3/3
3/3
d
60d RNAi
control
17/17
equinox RNAi
18/18
90d RNAi
control
17/17
equinox RNAi
18/18
e
control
equinox RNAi
0h
3h
6h
16h
24h
48h
dorsal markers
ventral markers

Figure S10

Supplementary Figure 10. *equinox* is not required for normal homeostasis and does not affect the DV axis. (a) 14 dpa blastemas are still not observed at anterior-facing wounds, but morphallaxis is observed at posterior-facing wounds associated with a new posterior pole in *equinox* RNAi animals. (b) Normal tissue turnover in control animals. (c) Epidermis covers the wound in *equinox* RNAi animals. (d) No defects are observed in long-term *equinox* RNAi animals. (e) Heatmap shows no ventralization after *equinox* RNAi. Live images are representative of three independent experiments. FISH images are representative of two independent experiments. Scale bars, 100 μm.

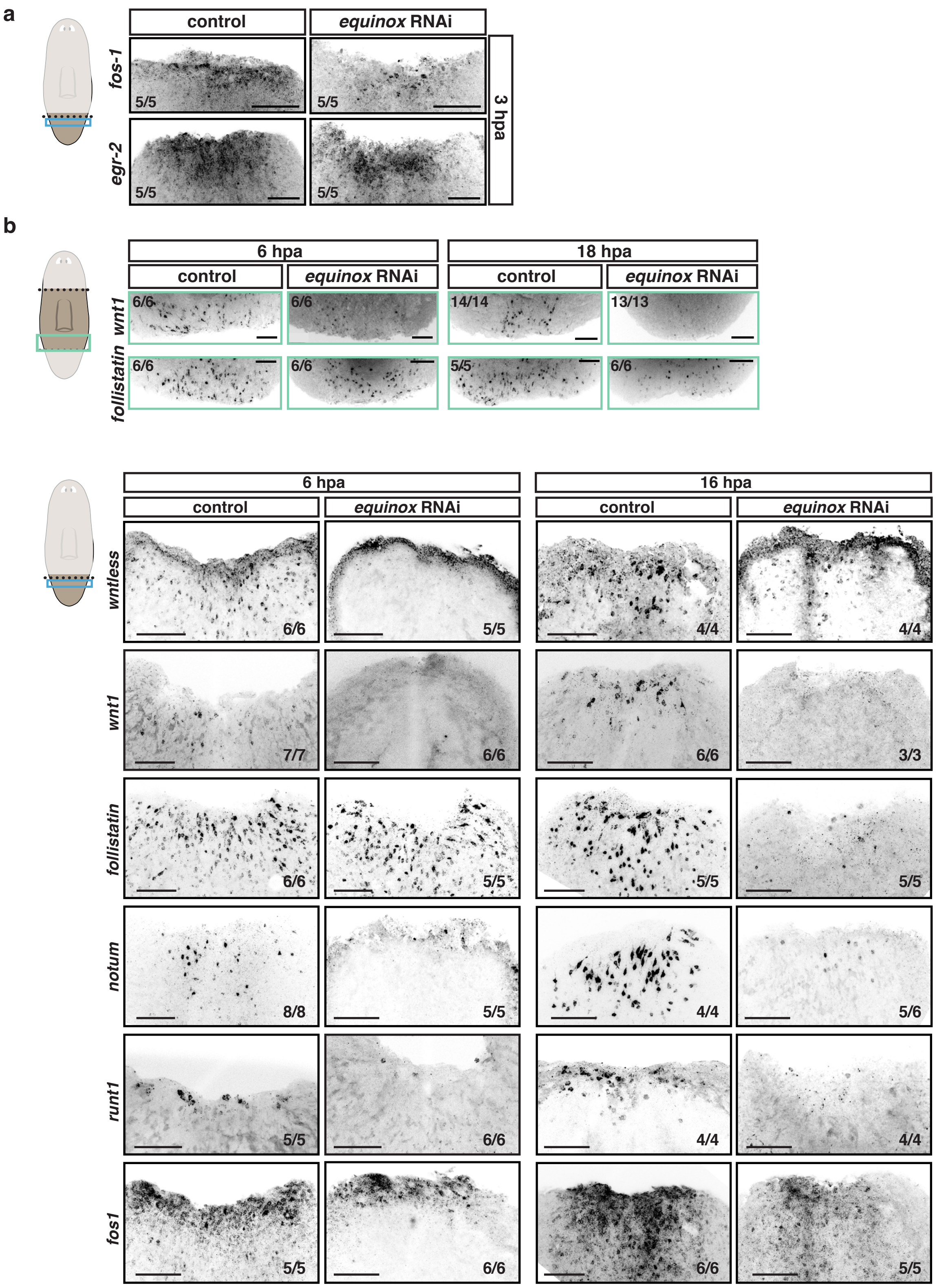
a
control
equinox RNAi
fos-1
egr-2
5/5
3 hpa
b
6 hpa
18 hpa
wnt1
follistatin
6/6
14/14
13/13
5/5
16 hpa
wntless
notum
runt1
fos1
7/7
8/8
4/4
3/3
5/6

Figure S11

Supplementary Figure 11. *equinox* is required for maintenance of wound-induced gene expression. (a) Activation of early wound-induced genes but (b) failure to maintain muscle wound-induced gene expression in *equinox* RNAi animals. Images shown are representative of two independent experiments. Scale bars, 100 μm.

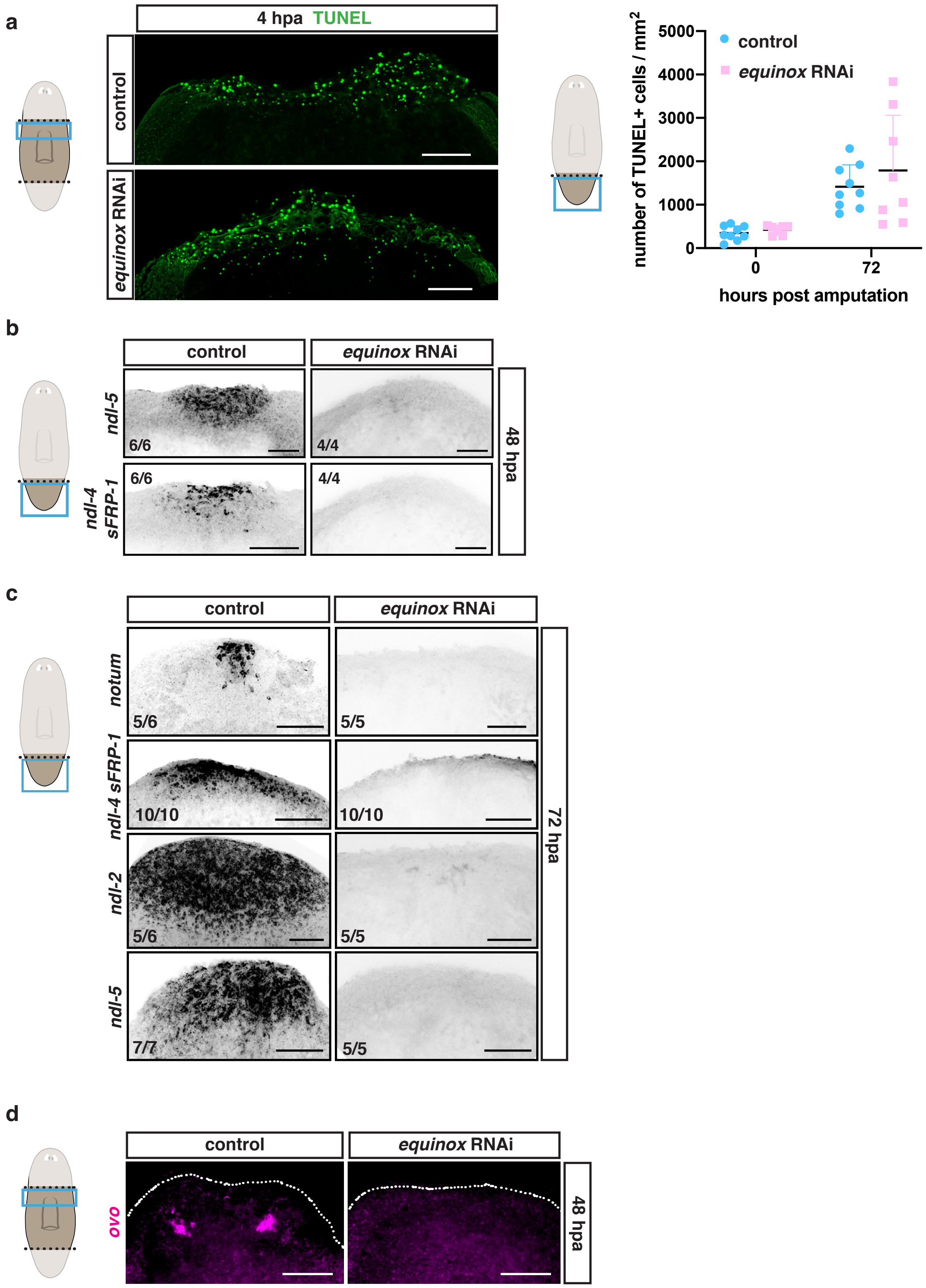
a
4 hpa TUNEL
control
equinox RNAi
number of TUNEL+ cells / mm2
5000
4000
3000
2000
1000
0
control
equinox RNAi
0
72
hours post amputation
b
control
equinox RNAi
ndl-5
ndl-4
sFRP-1
6/6
4/4
6/6
4/4
48 hpa
c
control
equinox RNAi
notum
ndl-4 sFRP-1
ndl-2
ndl-5
5/6
5/5
10/10
10/10
5/6
5/5
7/7
5/5
72 hpa
d
control
equinox RNAi
ovo
48 hpa

Figure S12

Supplementary Figure 12. Normal apoptosis response but no PCG rescaling in *equinox* RNAi animals. (a) *equinox* RNAi animals show similar numbers of TUNEL+ cells than control animals. (b, c) No expression of anterior PCGs after *equinox* RNAi at 48 hpa (b) or 72 hpa (c). For TUNEL experiments, n=9 independent animals for control 0 hpa and 72 hpa, and n=7 independent animals for *equinox* RNAi 0 hpa and n=8 for 72 hpa over one experiment. Data are presented as mean ± SD and analyzed with unpaired Student's t test. (d) No differentiation of eye cells after *equinox* RNAi. All FISH images are representative of two independent experiments. Source data are provided as a Source Data file. Scale bars, 100 μm.

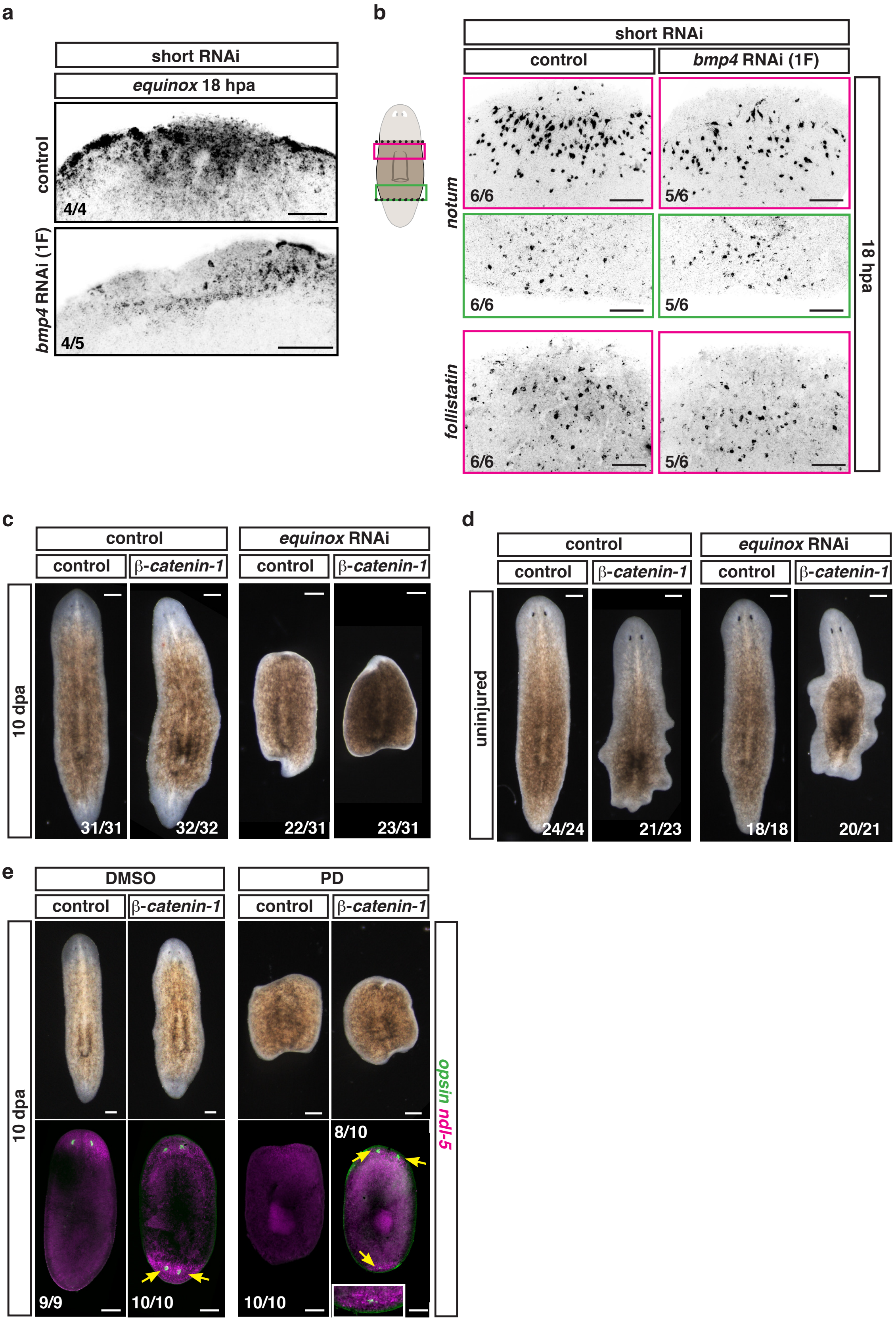
a
short RNAi
equinox 18 hpa
control
4/4
bmp4 RNAi (1F)
4/5
b
short RNAi
control
bmp4 RNAi (1F)
notum
6/6
5/6
6/6
5/6
follistatin
6/6
5/6
18 hpa
c
control
equinox RNAi
control
β-catenin-1
control
β-catenin-1
10 dpa
31/31
32/32
22/31
23/31
d
control
equinox RNAi
control
β-catenin-1
control
β-catenin-1
uninjured
24/24
21/23
18/18
20/21
e
DMSO
PD
control
β-catenin-1
control
β-catenin-1
10 dpa
opsin ndl-5
9/9
10/10
10/10
8/10

Figure S13

Supplementary Figure 13. *equinox* is required for blastema formation. Reduced expression of *equinox* (a) but normal wound-induced gene expression (b) at 18 hpa after only one *bmp4* RNAi feeding. (c) Inhibition of *β-catenin-1* does not suppress lack of blastema formation in *equinox* RNAi animals, however, (d) peripheral ectopic heads can be observed after inhibition of both genes in uninjured animals. (e) Inhibition of Erk signaling inhibits blastema formation but not PCG rescaling and differentiation of anterior tissues in *β-catenin-1* RNAi animals. Live images are representative of three independent experiments. FISH images are representative of two independent experiments.

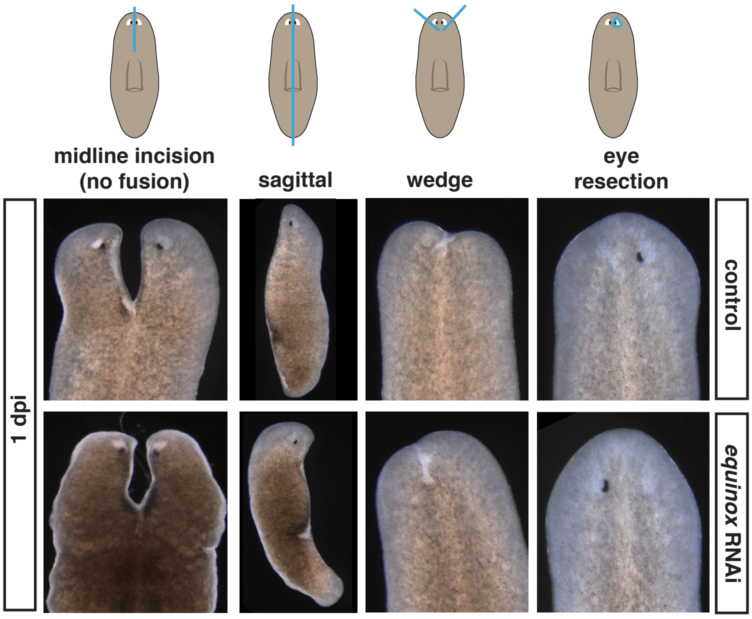
midline incision
(no fusion)
sagittal
wedge
eye
resection
1 dpi
control
equinox RNAi

Figure S14

Supplementary Figure 14. **Normal healing of diverse injuries after *equinox* RNAi.** *equinox* and control RNAi animals look alike one day post-injury (dpi). Live images are representative of three independent experiments. Scale bars, 100 µm.
