# Supplementary material for: The planarian wound epidermis gene equinox is required for blastema formation in regeneration
Source: Nat Commun. 2022 May 18;13:2726. doi: 10.1038/s41467-022-30412-6 (PMC9117669; doi:10.1038/s41467-022-30412-6)
Supplement: Supplementary file 3 — Description of Additional Supplementary Files [file 41467_2022_30412_MOESM3_ESM.pdf]

Description of Additional Supplementary Files

File Name: Supplementary Data 1
Description: **Differential expression analysis of regenerating control and *bmp4* RNAi animals.** The data provided here are calculated using the nbimontest function in DESeq. This is a one tailed binomial test. The padj column uses the Benjamini-Hochberg procedure to adjust for multiple tests.

File Name: Supplementary Data 2
Description: **Accession numbers of proteins with similar structure analyzed in the phylogenetic tree.**

File Name: Supplementary Data 3
Description: **Differential expression analysis of regenerating control and *equinox* RNAi animals.** The data provided here are calculated using the nbimontest function in DESeq. This is a one tailed binomial test. The padj column uses the Benjamini-Hochberg procedure to adjust for multiple tests.
