# Supplementary material for: The planarian wound epidermis gene equinox is required for blastema formation in regeneration
Source: Nat Commun. 2022 May 18;13:2726. doi: 10.1038/s41467-022-30412-6 (PMC9117669; doi:10.1038/s41467-022-30412-6)
Supplement: Supplementary file 7 — Reporting Summary [file 41467_2022_30412_MOESM7_ESM.pdf]

nature portfolio

Corresponding author(s): Peter Reddien

Last updated by author(s): Mar 2, 2022

Reporting Summary

Nature Portfolio wishes to improve the reproducibility of the work that we publish. This form provides structure for consistency and transparency in reporting. For further information on Nature Portfolio policies, see our Editorial Policies and the Editorial Policy Checklist.

Statistics

For all statistical analyses, confirm that the following items are present in the figure legend, table legend, main text, or Methods section.

n/a	Confirmed	
☐	☒	The exact sample size (*n*) for each experimental group/condition, given as a discrete number and unit of measurement
☐	☒	A statement on whether measurements were taken from distinct samples or whether the same sample was measured repeatedly
☐	☒	The statistical test(s) used AND whether they are one- or two-sided<br>*Only common tests should be described solely by name; describe more complex techniques in the Methods section.*
☐	☒	A description of all covariates tested
☐	☒	A description of any assumptions or corrections, such as tests of normality and adjustment for multiple comparisons
☐	☒	A full description of the statistical parameters including central tendency (e.g. means) or other basic estimates (e.g. regression coefficient) AND variation (e.g. standard deviation) or associated estimates of uncertainty (e.g. confidence intervals)
☐	☒	For null hypothesis testing, the test statistic (e.g. *F*, *t*, *r*) with confidence intervals, effect sizes, degrees of freedom and *P* value noted<br>*Give P values as exact values whenever suitable.*
☐	☒	For Bayesian analysis, information on the choice of priors and Markov chain Monte Carlo settings
☒	☐	For hierarchical and complex designs, identification of the appropriate level for tests and full reporting of outcomes
☒	☐	Estimates of effect sizes (e.g. Cohen's *d*, Pearson's *r*), indicating how they were calculated

Our web collection on statistics for biologists contains articles on many of the points above.

Software and code

Policy information about availability of computer code

Data collection	No software was used for data collection.
Data analysis	Seurat 3.2.3. MrBayes 3.2.6 x64. DESeq 1. Muscle Alignment (as hosted on web service). Cell Ranger 4.0.0. Bowtie 1.2.2. ggplot2 3.3.5. pheatmap 1.0.12.

For manuscripts utilizing custom algorithms or software that are central to the research but not yet described in published literature, software must be made available to editors and reviewers. We strongly encourage code deposition in a community repository (e.g. GitHub). See the Nature Portfolio guidelines for submitting code & software for further information.

Data

Policy information about availability of data

All manuscripts must include a data availability statement. This statement should provide the following information, where applicable:

- Accession codes, unique identifiers, or web links for publicly available datasets
- A description of any restrictions on data availability
- For clinical datasets or third party data, please ensure that the statement adheres to our policy

The sequencing data generated in this study have been deposited in the GenBank database under accession codes (1) GSE179290 [Single cell gene expression profiling using 10x v3; https://www.ncbi.nlm.nih.gov/geo/query/acc.cgi?acc=GSE179290
], (2) GSE179291 [bmp4 RNAi gene expression profiling; https://www.ncbi.nlm.nih.gov/geo/query/acc.cgi?acc=GSE179291], (3)GSE179293 [equinox RNAi gene expression profiling; https://www.ncbi.nlm.nih.gov/geo/query/acc.cgi?acc=GSE179293], (4) OM864265
(equinox gene deposition). The anti-H3P labeling data and processed DESeq comparison data for bulk sequencing generated in this study are provided in the

Supplementary Information/Source Data file.

Field-specific reporting

Please select the one below that is the best fit for your research. If you are not sure, read the appropriate sections before making your selection.

☒ Life sciences ☐ Behavioural & social sciences ☐ Ecological, evolutionary & environmental sciences

For a reference copy of the document with all sections, see nature.com/documents/nr-reporting-summary-flat.pdf

Life sciences study design

All studies must disclose on these points even when the disclosure is negative.

Sample size	Sample size was determined for bulk RNA sequencing experiments after using previous data from Scimone et al 2017 (myoD data) and performing a analysis using the software, Scotty. This analysis estimated that we would reach 50% of true positive genes detected at at p value cutoff of 0.01 if we used five replicates at a sequencing depth of 1313.8 million reads per replicate. For FISH experiments, sample size was not determined prior to experiment.
Data exclusions	There were no data exclusions in any of the analyses.
Replication	All experimental findings were reproduced in at least 2 independent experiments.
Randomization	Animals for all experiments were randomly selected from a large collection of clonal animals.
Blinding	Investigators were not blinded during data collection and analysis. Investigators were not blinded as the nature of the phenotypes was visible to the naked eye; bmp4 RNAi animals demonstrated dorsalization, while both bmp4 and equinox RNAi animals did not regenerate.

Reporting for specific materials, systems and methods

We require information from authors about some types of materials, experimental systems and methods used in many studies. Here, indicate whether each material, system or method listed is relevant to your study. If you are not sure if a list item applies to your research, read the appropriate section before selecting a response.

Materials & experimental systems

n/a	Involved in the study	
☐	☒	Antibodies
☒	☐	Eukaryotic cell lines
☒	☐	Palaeontology and archaeology
☐	☒	Animals and other organisms
☒	☐	Human research participants
☒	☐	Clinical data
☒	☐	Dual use research of concern

Methods

n/a	Involved in the study	
☒	☐	ChIP-seq
☐	☒	Flow cytometry
☒	☐	MRI-based neuroimaging

Antibodies

Antibodies used	SMEDWI-1 antibody was used at 1:1000. Commercial primary antibodies used also included an anti-phospho-Histone H3 antibody (Millipore 05-817R-I, clone 63-1C-8; 1:300 in 5% inactivated horse serum)
Validation	SMEDWI-1 antibody was made as in Guo et al Developmental Cell 2006, and validated in Scimone et al Dev. and Stem Cells 2010. anti-phospho-Histone H3 antibody was previously described in Tewari et al Cell. Reports 2018, and Wenemoser et al Developmental Biology 2010; per manufacturer's website 'This highly published Ab, also known as Anti-H3S10p, has been validated in ICC, IP & WB.'

Animals and other organisms

Policy information about studies involving animals; ARRIVE guidelines recommended for reporting animal research

Laboratory animals	Schmidtea mediterranea ClW4, asexual strain. This strain was collected from Spain in the 1990s, and has been continually passaged since then. It is clonal and relies on fission for asexual reproduction.
Wild animals	No wild animals were used in this study.

Field-collected samples	No field collected samples were used in this study.
Ethics oversight	No ethical oversight or protocols were required as all animals used in this study were invertebrates.

Note that full information on the approval of the study protocol must also be provided in the manuscript.

Flow Cytometry

Plots

Confirm that:

☒ The axis labels state the marker and fluorochrome used (e.g. CD4-FITC).

☒ The axis scales are clearly visible. Include numbers along axes only for bottom left plot of group (a 'group' is an analysis of identical markers).

☒ All plots are contour plots with outliers or pseudocolor plots.

☒ A numerical value for number of cells or percentage (with statistics) is provided.

Methodology

Sample preparation	See methods for sample preparation. Cells were collected from primary tissue which was then macerated with a scalpel. Cells were then further dissociated in trypsin. They were labeled with Hoescht and/or PI and/or calcein. Negative and single label controls were included. Cells were sorted in a medial of PBS and low. percent BSA (0.1-1%).
Instrument	Aria FACS sorter.
Software	None.
Cell population abundance	We took 100% of live cells.
Gating strategy	We used the standard gating for the field as seen in Hayashi et al., 2006, and Reddien et al., 2005. This strategy can be grossly described by the following; take all events which are reasonably sized as live cells in FSC/SSC plot. Take the PI low and calcein med/high events to gate in all live cells. Take the lower branch of events (as shown in supplemental figure 5a) on the Hoescht plot to take cells with lower DNA content (not currently dividing).

☒ Tick this box to confirm that a figure exemplifying the gating strategy is provided in the Supplementary Information.
